# Inter-observer variability amongst surgeons and radiologists in assessment of Guy’s Stone Score and S.T.O.N.E. nephrolithometry score: A prospective evaluation

**DOI:** 10.1080/2090598X.2019.1703278

**Published:** 2019-12-18

**Authors:** Aneesh Srivastava, Priyank Yadav, Kumar Madhavan, Sanjoy K. Sureka, Uday P. Singh, Rakesh Kapoor, M.S. Ansari, Hira Lal, Prabhakar Mishra

**Affiliations:** a Department of Urology and Renal Transplantation, Sanjay Gandhi Postgraduate Institute of Medical Sciences, Lucknow, India; b Department of Radiology, Sanjay Gandhi Postgraduate Institute of Medical Sciences, Lucknow, India; c Department of Biostatistics, Sanjay Gandhi Postgraduate Institute of Medical Sciences, Lucknow, India

**Keywords:** Inter-observer variability, Guy’s Stone Score, S.T.O.N.E. nephrolithometry score

## Abstract

**Objective:**

(a) To assess the inter-observer variability amongst surgeons performing percutaneous nephrolithotomy (PCNL) and radiologists for the Guy’s Stone Score (GSS) and S.T.O.N.E. (stone size [S], tract length [T], obstruction [O], number of involved calyces [N], and essence or stone density [E]) nephrolithometry score; (b) To determine which scoring system of the two is better for predicting the stone-free rate (SFR) after PCNL.

**Patients, subjects and methods:**

Patients undergoing PCNL between February 2016 and September 2016 were prospectively enrolled. Preoperative computed tomography was done in all patients. The GSS and S.T.O.N.E. nephrolithometry score were independently calculated by eight surgeons and four radiologists. The patients were operated on by one of the surgeons (all were consultants). The Fleiss’ κ coefficient was used to assess agreement independently between the surgeons and radiologists. Receiver operating characteristic (ROC) curves were constructed for predicting the SFR using the average of the scores of the surgeons and radiologists separately.

**Results:**

A total of 157 patients underwent PCNL. The SFR was 71.3% (112/157 patients). The Fleiss’ κ scores ranged from 0.51 to 0.88 (overall 0.79) for the S.T.O.N.E. score and 0.53–0.91 for the GSS, suggesting moderate to very good agreement. The ROC curve for the S.T.O.N.E. nephrolithometry scores of surgeons (area under the curve [AUC] = 0.806) as well as the radiologists (AUC = 0.810) had a higher predictive value for the SFR than the GSS of the surgeons (AUC = 0.738) and the radiologists (AUC = 0.747).

**Conclusion:**

There is overall good agreement between surgeons and radiologists for both the GSS and S.T.O.N.E. nephrolithometry score. The S.T.O.N.E. score had a higher predictive value for the SFR than the GSS.

**Abbreviations:**

AUC: area under the curve; GSS: Guy’s Stone Score; KUB: kidneys, ureters and bladder; NCCT: non-contrast CT; PCNL: Percutaneous nephrolithotomy; ROC: receiver operating characteristic; SFR: stone-free rate; S.T.O.N.E.: stone size [S], tract length [T], obstruction [O], number of involved calyces [N], and essence or stone density [E]

## Introduction

Percutaneous nephrolithotomy (PCNL) is one of the commonest surgical treatments offered to patients with renal calculi. As the technology has advanced over the last three decades, miniaturisation of instruments has been possible, which has resulted in a decrease in the perioperative morbidity associated with PCNL [1]. However, since the procedure is inherently invasive and requires puncture and dilatation of the renal parenchyma, complications such as bleeding requiring transfusion, urine extravasation, and adjacent organ injury, continue to occur in some patients. Having reached the limits of miniaturisation, the recent approach to the prevention of PCNL-related morbidity is prediction of patients at risk of such complications. Various scoring systems have been proposed to grade patients according to complexity of the stone and pelvicalyceal system [2–5].

The main drawback of these systems is that none of them is perfect. Most of the published studies have focussed on re-validating them or comparing these systems with one another on the basis of individual parameters, ability to predict the stone-free rate (SFR), and complications [6–18]. Few have attempted to compare the agreement between different groups of trainees and surgeons [5,19]. The purpose of a scoring system is to act as a tool to predict the difficulty level, success rate, and complications of the surgery it is used for. In the case of PCNL, the existing scoring systems have variably predicted these. Another important feature of a scoring system is to bridge the communication gap between different departments working on the same pathology. The use of the Gleason grade for prostate cancer is the best example, where the pathologist and urologist have the same interpretation of a particular value of the score [20]. Similarly, grading of hydronephrosis by the Society for Fetal Urology also has good concordance amongst the radiologist and urologist [21]. Such a level of agreement has not been established for imaging of renal calculi; hence, we sought to determine if two of the commonest scoring systems used for renal calculi (the Guy’s Stone Score [GSS]; and the S.T.O.N.E., stone size [S], tract length [T], obstruction [O], number of involved calyces [N], and essence or stone density [E], nephrolithometry score) are interpreted in a similar way by the radiologist and urologist, and which one better predicts the SFR.

## Patients, subjects and methods

This study was performed at a tertiary care centre in northern India. Patients undergoing PCNL between February 2016 and September 2016 were prospectively enrolled in this study. For calculation of sample size, a power of 0.8 and an α of 0.05 were selected. Apart from this, chance agreement amongst the raters was the null hypothesis and was set at a κ of 0.4. As 0.8 was the minimum value needed for very good agreement, this was taken as the expected value [22]. This led us to a sample size of 156. Preoperative non-contrast CT (NCCT) was done in all patients. Patients aged <18 years, a history of prior surgery on the ipsilateral kidney, and nephrostomy tube or stent placement in the ipsilateral kidney prior to surgery, were excluded from the study. For the patients who were included in the study, the GSS [2] and S.T.O.N.E. nephrolithometry score [3] were independently calculated by eight surgeons (five consultants and three residents) and four radiologists (two consultants and two residents). Every patient was operated on by one of the five consultant surgeons (A.S., S.K.S., R.K., U.P.S., M.S.A.).

At the time of PCNL, a ureteric catheter was placed routinely. Percutaneous access to the pelvicalyceal system was established under fluoroscopic guidance using the ‘Bull’s eye’ technique. The tract was dilatated to 16–30 F depending upon the stone burden. Stone fragmentation was done using a pneumatic lithoclast or holmium laser. After the procedure, a JJ stent was placed routinely, while nephrostomy was placed in most cases. Success was defined as absence of radio-opaque shadow on postoperative plain abdominal radiograph of the kidneys, ureters and bladder (KUB, on first day after surgery) or absence of stone density on CT (for radiolucent calculi after JJ stent removal). The demographic characteristics, presence of residual stones, operating time, hospital stay, and fluoroscopy time were recorded. The complications were graded as per the modified Clavien–Dindo classification.

Data were first checked for normal distribution using the Shapiro–Wilk test. Parametric data were described using the mean ± standard deviation (SD) and non-parametric data using the median and range. Further, the statistical significance of variables with respect to the GSS (four categories) and S.T.O.N.E. score (three categories) was tested using the chi-squared test for categorical variables (Clavien–Dindo Grade and stone-free status) and ANOVA for parametric continuous data (operating time and postoperative hospital stay).

GSS

- GSS 1: a solitary stone in the mid and/or lower pole or in the renal pelvis with normal anatomy.
- GSS 2: a solitary stone in the upper pole; multiple stones in a patient with simple anatomy; or a solitary stone in a patient with abnormal anatomy.
- GSS 3: multiple stones in a patient with abnormal anatomy or in a calyceal diverticulum or partial staghorn calculus (defined as a stone involving the renal pelvis and at least two calyces).
- GSS 4: a complete staghorn calculus (all calyces and the pelvis occupied by stones) or any stone in a patient with spina bifida or a spinal injury.

S.T.O.N.E. score

- Takes into account stone size, tract length, obstruction, number of calyces and stone essence (in Hounsfield units [HU]).
- Minimum score is 3 and maximum score is 13.
- Low complexity is indicated by a score of 5–6, moderate complexity by a score of 7–8, and high complexity by a score of 9–13.

Fleiss’ κ coefficient was used to assess agreement independently between the two groups (surgeons vs radiologists). The following κ categories were used [23]:

- <0.20: Poor agreement
- 0.20–0.40: Fair agreement
- 0.41–0.60: Moderate agreement
- 0.61–0.80: Good agreement
- 0.81–1.00: Very good agreement

To evaluate which scoring system better predicted the SFR; four receiver operating characteristic (ROC) curves were drawn using the average scores of the surgeons and radiologists for the GSS and S.T.O.N.E. score for each patient. The area under the curve (AUC) of the ROC curve was compared using the DeLong method [24]. The SFR was defined as the presence of stone fragments of ≤4 mm. The data analysis was done using the IBM Statistical Package for the Social Sciences (SPSS®) for Mac, version 23 (SPSS Inc., IBM Corp., Armonk, NY, USA.); and MedCalc Statistical Software, version 18.2.1 (MedCalc Software, Ostend, Belgium).

## Results

A total of 157 patients underwent PCNL, of which 102 were men (65%) and 55 were women (34%). The mean (SD) age of the patients was 38.2 (13.4) years. The mean (SD) operating time was 122 (35) min, the mean (SD) fluoroscopy time was 6.8 (2.6) min, and the mean (SD) hospital stay was 3.4 (1.3) days. Postoperative complications were categorised in accordance with the modified Clavien–Dindo classification, 124 (78.9%) patients were considered as Grade 0 (normal postoperative trajectory without any unexpected deviation), 20 (12.7%) as Grade I (fever, pain management with NSAIDs), 11 (7.0%) as Grade II (fever treated with antibiotics, bleeding requiring blood transfusion), and two (1.2%) as Grade III (renal pelvic perforation managed by ureteric stenting, pleural effusion requiring intercostal drainage). The operative variables of the patients are listed in Table 1.

**Table 1.** Operative variables.

Variable	Value
Total number of patients	157
Male:female, *n*	1.85:1
Age, years, mean (SD)	38.2 (13.4)
Right/left, *n*	83/74
Operating time, min, mean (SD)	122.7 (35.2)
Fall in Hb (preoperative – postoperative value), g/dL, mean (SD)	1.4 (0.5)
Hospital stay, days, mean (SD)	3.4 (1.3)
Complications, *n* (%)	
Clavien–Dindo Grade I	20 (12.7)
Clavien–Dindo Grade II	11 (7.0)
Clavien–Dindo Grade III	2 (1.2)
SFR, *n* (%)	112 (71.3)

Hb, haemoglobin.

According to the KUB radiograph for radio-opaque calculi (*n* = 126) and NCCT KUB for radiolucent calculi (*n* = 31), as described in the Methods, 112 patients were found to be residual stone free (71.3%; 89 radio-opaque and 23 radiolucent) and 45 patients (28.7%; 37 radio-opaque and eight radiolucent) had residual stones after a single procedure. According to the GSS, 70% (*n* = 110) of the patients were GSS 1, 21.6% (*n* = 34) GSS 2, 7% (*n* = 11) GSS 3, and 1.27% (*n* = 2) GSS 4. The median (range) S.T.O.N.E. score was 7 (3–13). Further, using the S.T.O.N.E. score, patients were classified as having low (*n* = 98; 62.42%), moderate (*n* = 44; 28.03%), and high (*n* = 15; 9.55%) complexity stone disease. In this study, 86.3% of GSS 1, 44.2% of GSS 2, and 18.1% of GSS 3 patients had stone clearance (Table 2). According to the S.T.O.N.E. score categories; 90.8% of the patients with a S.T.O.N.E. score of 5–6 (low complexity), 41% with a S.T.O.N.E. score of 7–8 (moderate complexity), and 33.3% with a S.T.O.N.E. score of 9–13 (high complexity) had stone clearance (Table 2).

**Table 2.** SFR according to the GSS and S.T.O.N.E. score.

Scoring system	SFR, *n* (%)	Complications, *n*
**GSS**		
GSS 1 (*n* = 110)	95 (86.3)	18
GSS 2 (*n* = 34)	15 (44.2)	9
GSS 3 (*n* = 11)	2 (18.1)	4
GSS 4 (*n* = 2)	0	2
Total (*n* = 157)	112 (71.3)	33
**S.T.O.N.E. score**		
Low complexity, 5–6 (*n* = 98)	89 (90.8)	8
Moderate complexity, 7–8 (*n* = 44)	18 (41)	11
High complexity, 9–13 (*n* = 15)	5 (33.3)	14
Total (*n* = 157)	112 (71.3)	33

There was a statistically significant association between the total S.T.O.N.E. score and the Clavien–Dindo Grade (*P* < 0.001), operating time (*P* = 0.012), and stone-free status (*P* < 0.001). A statistically significant association was also found between the GSS and the Clavien–Dindo Grade (*P* < 0.001), hospital stay (*P* < 0.001), operating time (*P* < 0.001), and SFR (*P* = 0.05).

Inter-rater agreement was studied between the surgeons and radiologists. It was found that for the S.T.O.N.E. score (overall good agreement 0.79), tract length had very good agreement, whilst obstruction had only moderate agreement (Table 3). The remaining parameters had good agreement. For the GSS, there was very good agreement for GSS 1 and GSS 4, whilst GSS 2 and GSS 3 had moderate and good agreement, respectively. The categorisation of pelvicalyceal anatomy as ‘normal’ or ‘abnormal’ was the commonest point of contention between the radiologists and the surgeons.

**Table 3.** Fleiss’ κ coefficient for inter-rater agreement between the surgeons (eight) and the radiologists (four) for the S.T.O.N.E. score and GSS.

Assessment criterion	Agreement	Fleiss’ κ coefficient
**S.T.O.N.E.**		
S, stone size (*n* = 157)	Good	0.75
T, tract length (*n* = 157)	Very good	0.88
O, obstruction (*n* = 157)	Moderate	0.51
N, number of involved calices (*n* = 157)	Good	0.80
E, essence or stone density (*n* = 157)	Good	0.78
Overall (*n* = 157)	Good	0.79
**GSS**		
GSS 1 (*n* = 110)	Very good	0.91
GSS 2 (*n* = 34)	Moderate	0.53
GSS 3 (*n* = 11)	Good	0.61
GSS 4 (*n* = 2)	Very good	0.84

**Figure 1.**
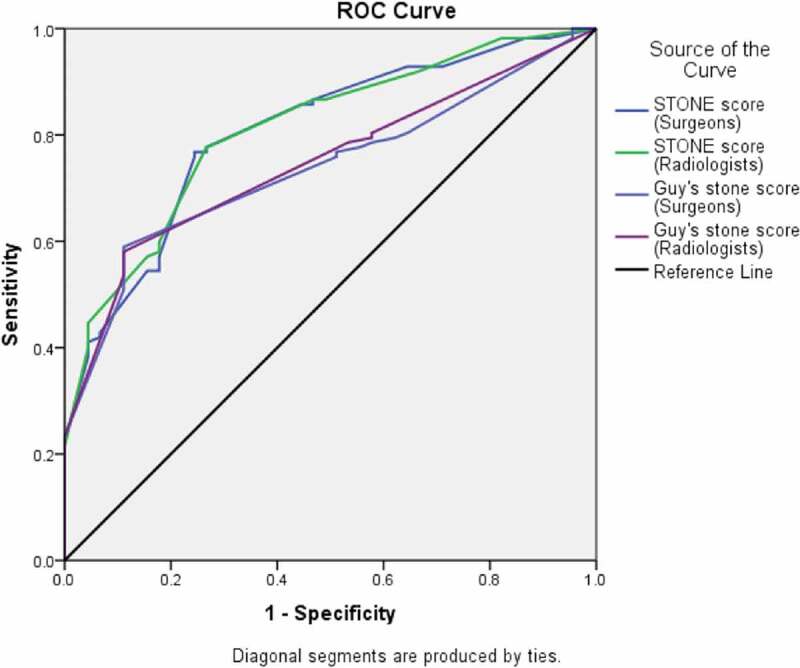
ROC curve analysis of the surgeons’ and radiologists’ GSSs and S.T.O.N.E. scores.

ROC curve analysis revealed that the S.T.O.N.E. nephrolithometry score of the surgeons (AUC = 0.806), as well as the radiologists (AUC = 0.810) had a higher AUC as compared to the GSS of the surgeons (AUC = 0.738) and the radiologists (AUC = 0.747) (Figure 1). Using the DeLong method, the S.T.O.N.E. score of surgeons better predicted the SFR than their GSS (SE 0.0302, 95% CI 0.0088–0.127, *z*-score 2.252; *P* = 0.024). A similar result was obtained for the radiologists scores (SE 0.0269, 95%CI 0.0104–0.116, *z*- score 2.346; *P* = 0.019).

## Discussion

Previously, it has been shown that the scoring systems in use are good predictors of complications following PCNL [2–5]. There is also acceptable reproducibility of these scores amongst different surgeons [14–18]. However, whether there is any difference in the interpretation of these scoring systems amongst radiologists, who report the CT of these patients but are not involved in PCNL, is not addressed well in the literature. This is important especially for the centres where the percutaneous access to the kidney is established by the radiologist. The variability in the grading of the GSS and S.T.O.N.E. scoring systems was assessed when the same system was used by junior and senior surgeons and radiologists. It was found that the S.T.O.N.E. nephrolithometry score fares better in terms of reproducibility amongst these groups compared to the GSS. Further, parameters amongst these systems that have the maximum discordance were also identified.

The GSS is primarily based on the number/location of the stone and anatomy of the kidney. The greatest contention occurs with respect to the interpretation of ‘abnormal’ anatomy. The authors described abnormal anatomy as ‘abnormal renal and/or collecting system anatomy, or presence of an ileal conduit’ and this does little to abate confusion. Most studies based on this scoring system report difficulties in assigning patients GSS 2 or 3, whereas GSS 1 and 4 are usually easy to assign. Another issue that is commonly reported with this scoring system is the lack of definition of a partial staghorn calculus [25]. On applying κ statistics, the highest correlation was found for GSS 1 followed by 4. There was discordance amongst the surgeons and radiologists for the assignment of GSS 2/3, and the factor which was most discordant was the presence of abnormal anatomy.

The S.T.O.N.E. score was proposed by Okhunov et al. [3] and includes easily measurable variables on NCCT to predict the outcomes of PCNL. This system is more objective and hence theoretically less prone to inter-observer variation. However, discordance was found in the reporting of hydronephrosis amongst the surgeons and radiologists. Although there are only two categories of hydronephrosis in this system, which simplifies the categorisation, the interpretation of pelvicalyceal morphology by the surgeons differed from the radiologists in several cases. What appeared as a ‘normal’ variation to the radiologist (such as an extrarenal pelvis) was often regarded as mild or even moderate hydronephrosis by the surgeon. Another observation was that the kidneys that were hydronephrotic were easier to puncture during PCNL and with a larger working space inside stone fragmentation and retrieval was easy too. Further, it was easier to access other calyces in a hydronephrotic kidney compared to a non-hydronephrotic kidney. While a lower agreement was found in the parameter of obstruction, Okhunov et al. [19] found that quantification of stone size and the number of calyces was least reliable. Compared to other systems, this system is an easy to use and reproducible scoring system particularly for observers across different specialties and with different levels of experience, making it a versatile system.

In the present study, 71.3% of the patients were stone free after PCNL. Other studies comparing the scoring systems for renal calculi have reported stone clearance rates after PCNL of between 62% and 90% [2–5,14–18]. The present study produced a comparable SFR. There was a significant relationship between both scoring systems and stone-free status. The GSS was externally validated by Mandal et al. [6] and Ingimarsson et al. [10], and they have reported it to be a reliable predictor of stone-free status. Vicentini et al. [8], as well as Sfoungaristos et al. [9], found the GSS to significantly predict the SFR. The initial SFRs reported by Mandal et al. [6], Ingimarsson et al. [10] and Vicentini et al. [8] were between 71% and 76%, whilst that reported by Sfoungaristos et al. [9] was 84.7%. In this study, there were a limited number of patients with complex grades (GSS 3 and 4).

Few studies have done direct comparisons of the various scoring systems. The GSS and S.T.O.N.E. scoring systems were compared by Noureldin et al. [14] and Kumsar et al. [15]. While Noureldin et al. [14] found that neither of them was superior to the other for predicting SFR; Kumsar et al. [15] reported that the S.T.O.N.E. score correlated well with the SFR but not complications, and the GSS correlated with none. Similarly, Bozkurt et al [16] compared the GSS with the Clinical Research Office of the Endourological Society (CROES) nephrolithometry nomogram and concluded that both were equally good and correlated with the SFR. Labadie et al. [17] and Tailly et al. [18] compared the GSS, S.T.O.N.E. score and CROES systems. Labadie et al. [17] concluded that only the GSS and S.T.O.N.E. scores correlated with complications, whereas Tailly et al. [18] found that none of them correlated with complications. Tailly et al. [18] also found that all three could predict residual fragments and were equivalent; this was also observed by Singla et al [26]. Both of these authors found S.T.O.N.E. scores to be overall superior and the easiest to apply.

The authors accept that the present study is limited by a relatively small number of patients compared to the burden of stone disease and being a single-centre study from a referral institute there is lack of generalisability to a larger population. However, the primary aim of the present study was determine the reproducibility of the GSS and S.T.O.N.E. score amongst surgeons and radiologists, and this was done prospectively, removing the potential bias that could happen amongst the surgeons after knowing the final outcome of surgery. Additionally, the study had some heterogeneity due to multiple operating surgeons (five), multiple techniques of dilatation, and the different methods of evaluating the SFR that were utilised. However, all the surgeons used standardised techniques of both conventional and mini PCNL. Although CT scan would have been the most accurate method for evaluation of the SFR, the additional exposure of radiation would have been unethical and was hence avoided in patients with radio-opaque stones.

## Conclusion

The overall agreement between the surgeons and radiologists for scoring patients with renal calculi as per the GSS and S.T.O.N.E. nephrolithometry score was good. The S.T.O.N.E. nephrolithometry score had a higher predictive value for the SFR than the GSS.

## References

[1] Mishra S, Sharma R, Garg C, et al. Prospective comparative study of miniperc and standard PNL for treatment of 1 to 2 cm size renal stone. BJU Int. 2011;108:896–899. 21477212 10.1111/j.1464-410X.2010.09936.x

[2] Thomas K, Smith NC, Hegarty N, et al. The Guy’s Stone Score - grading the complexity of percutaneous nephrolithotomy procedures. Urology. 2011;78:277–281. 21333334 10.1016/j.urology.2010.12.026

[3] Okhunov Z, Friedlander JI, George AK, et al. S.T.O.N.E. nephrolithometry: novel surgical classification system for kidney calculi. Urology. 2013;81:1154–1159. 23540858 10.1016/j.urology.2012.10.083

[4] Smith A, Averch TD, Shahrour K, et al. A nephrolithometric nomogram to predict treatment success of percutaneous nephrolithotomy. J Urol. 2013;190:149–156. 23353048 10.1016/j.juro.2013.01.047

[5] Jeong CW, Jung JW, Cha WH, et al. Seoul national university renal stone complexity score for predicting stone-free rate after percutaneous nephrolithotomy. PLoS ONE. 2013;8:e65888. 23824752 10.1371/journal.pone.0065888 PMC3688830

[6] Mandal S, Goel A, Kathpalia R, et al. Prospective evaluation of complications using the modified Clavien grading system, and of success rates of percutaneous nephrolithotomy using Guy’s Stone Score: a single-center experience. Indian J Urol. 2012;28:392–398. 23450640 10.4103/0970-1591.105749 PMC3579117

[7] Sinha RK, Mukherjee S, Jindal T, et al. Evaluation of stone-free rate using Guy’s Stone Score and assessment of complications using modified Clavien grading system for percutaneous nephrolithotomy. Urolithiasis. 2015;43:349–353. 25850962 10.1007/s00240-015-0769-1

[8] Vicentini FC, Marchini GS, Mazzucchi E, et al. Utility of the Guy’s Stone Score based on computed tomographic scan findings for predicting percutaneous nephrolithotomy outcomes. Urology. 2014;83:1248–1253. 24612615 10.1016/j.urology.2013.12.041

[9] Sfoungaristos S, Lorber A, Gofrit ON, et al. External validation and predictive accuracy assessment of Guy’s Stone Score as a preoperative tool for estimating percutaneous nephrolithotomy outcomes. J Endourol. 2015;29:1131–1135. 25936386 10.1089/end.2015.0273

[10] Ingimarsson JP, Dagrosa LM, Hyams ES, et al. External validation of a preoperative renal stone grading system: reproducibility and inter-rater concordance of the Guy’s Stone Score using preoperative computed tomography and rigorous postoperative stone-free criteria. Urology. 2014;83:45–49. 24210568 10.1016/j.urology.2013.09.008

[11] Noureldin YA, Elkoushy MA, Andonian S. External validation of the S.T.O.N.E. nephrolithometry scoring system. Can Urol Assoc J. 2015;9:190–195. 26225168 10.5489/cuaj.2652 PMC4479640

[12] Akhavein A, Henriksen C, Syed J, et al. Prediction of single procedure success rate using S.T.O.N.E. nephrolithometry surgical classification system with strict criteria for surgical outcome. Urology. 2015;85:69–73. 25530366 10.1016/j.urology.2014.09.010

[13] Choo MS, Jeong CW, Jung JH, et al. External validation and evaluation of reliability and validity of the S-ReSC scoring system to predict stone-free status after percutaneous nephrolithotomy. PLoS ONE. 2014;9:e83628. 24421896 10.1371/journal.pone.0083628 PMC3885452

[14] Noureldin YA, Elkoushy MA, Andonian S. Which is better? Guy’s versus S.T.O.N.E. nephrolithometry scoring systems in predicting stone-free status post-percutaneous nephrolithotomy. World J Urol. 2015;33:1821–1825. 25678344 10.1007/s00345-015-1508-5

[15] Ş K, Aydemir H, Halis F, et al. Value of preoperative stone scoring systems in predicting the results of percutaneous nephrolithotomy. Cent European J Urol. 2015;68:353–357. 10.5173/ceju.2015.552 PMC4643700 26568881

[16] Bozkurt IH, Aydogdu O, Yonguc T, et al. Comparison of guy and clinical research office of the endourological society nephrolithometry scoring systems for predicting stone-free status and complication rates after percutaneous nephrolithotomy: a single center study with 437 cases. J Endourol. 2015;29:1006–1010. 26153844 10.1089/end.2015.0199

[17] Labadie K, Okhunov Z, Akhavein A, et al. Evaluation and comparison of urolithiasis scoring systems used in percutaneous kidney stone surgery. J Urol. 2015;193:154–159. 25088952 10.1016/j.juro.2014.07.104

[18] Tailly TO, Okhunov Z, Nadeau BR, et al. Multicenter external validation and comparison of stone scoring systems in predicting outcomes after percutaneous nephrolithotomy. J Endourol. 2016;30:594–601. 26728427 10.1089/end.2015.0700

[19] Okhunov Z, Helmy M, Perez-lansac A, et al. Interobserver reliability and reproducibility of s.T.o.N.e. Nephrolithometry for renal calculi. J Endourol. 2013;27:1303–1306. 23815088 10.1089/end.2013.0289

[20] Billis A, Guimaraes MS, Freitas LL, et al. The impact of the 2005 international society of urological pathology consensus conference on standard Gleason grading of prostatic carcinoma in needle biopsies. J Urol. 2008;180:548–552. 18550106 10.1016/j.juro.2008.04.018

[21] Keays MA, Guerra LA, Mihill J, et al. Reliability assessment of society for fetal urology ultrasound grading system for hydronephrosis. J Urol. 2008;180(Suppl):1680–1682. 18708207 10.1016/j.juro.2008.03.107

[22] Bujang MA, Baharum N. A simplified guide to determination of sample size requirements for estimating the value of intraclass correlation coefficient: a review. Arch Orofac Sci. 2017;12:1–11.

[23] DeLong ER, DeLong DM, Clarke-Pearson DL. Comparing the areas under two or more correlated receiver operating characteristic curves: a nonparametric approach. Biometrics. 1988;44:837–845. 3203132

[24] McHugh ML. Interrater reliability: the kappa statistic. Biochem Med (Zagreb). 2012;22:276–282. 23092060 PMC3900052

[25] Wu WJ, Okeke Z. Current clinical scoring systems of percutaneous nephrolithotomy outcomes. Nat Rev Urol. 2017;14:459–469. 28534536 10.1038/nrurol.2017.71

[26] Singla A, Khattar N, Nayyar R, et al. How practical is the application of percutaneous nephrolithotomy scoring systems? Prospective study comparing Guy’s Stone Score, S.T.O.N.E. score and the clinical research office of the endourological society (CROES) nomogram. Arab J Urol. 2017;15:7–16. 28275512 10.1016/j.aju.2016.11.005 PMC5329720

